# Prediction of type 2 diabetes mellitus based on nutrition data

**DOI:** 10.1017/jns.2021.36

**Published:** 2021-06-21

**Authors:** Andreas Katsimpris, Aboulmaouahib Brahim, Wolfgang Rathmann, Anette Peters, Konstantin Strauch, Antònia Flaquer

**Affiliations:** 1 Chair of Genetic Epidemiology, Institute of Medical Informatics, Biometry and Epidemiology, Ludwig-Maximilians-Universität, Munich, Germany; 2 Institute for Medical Informatics, Biometry and Epidemiology (IBE), Ludwig-Maximilians-Universität, Munich, Germany; 3 Department of Biometry and Epidemiology, German Diabetes Center, Düsseldorf, Germany; 4 Helmholtz Zentrum München, German Research Center for Environmental Health, Institute of Epidemiology II, Neuherberg, Munich, Germany; 5 Institute of Medical Biostatistics, Epidemiology and Informatics (IMBEI), University Medical Center, Johannes Gutenberg University, Mainz, Germany

**Keywords:** Elastic net regression, Nutrition, Prediction model, Type 2 diabetes, 24HFL, 24-h food list, KORA, Cooperative Health Research in the Region of Augsburg, NPV, negative predictive value, PPV, positive predictive value, ROC, receiver operating characteristic, T2DM, type 2 diabetes mellitus

## Abstract

Numerous predictive models for the risk of type 2 diabetes mellitus (T2DM) exist, but a minority of them has implemented nutrition data so far, even though the significant effect of nutrition on the pathogenesis, prevention and management of T2DM has been established. Thus, in the present study, we aimed to build a predictive model for the risk of T2DM that incorporates nutrition data and calculates its predictive performance. We analysed cross-sectional data from 1591 individuals from the population-based Cooperative Health Research in the Region of Augsburg (KORA) FF4 study (2013–14) and used a bootstrap enhanced elastic net penalised multivariate regression method in order to build our predictive model and select among 193 food intake variables. After selecting the significant predictor variables, we built a logistic regression model with these variables as predictors and T2DM status as the outcome. The values of area under the receiver operating characteristic (ROC) curve, sensitivity, specificity, positive predictive value (PPV), negative predictive value (NPV) and accuracy of our predictive model were calculated. Eleven out of the 193 food intake variables were selected for inclusion in our model, which yielded a value of area under the ROC curve of 0⋅79 and a maximum PPV, NPV and accuracy of 0⋅37, 0⋅98 and 0⋅91, respectively. The present results suggest that nutrition data should be implemented in predictive models to predict the risk of T2DM, since they improve their performance and they are easy to assess.

## Introduction

Type 2 diabetes mellitus (T2DM) is a chronic metabolic disorder associated with numerous complications, which can affect multiple organ systems^(1)^. T2DM is also recognised as a serious, worldwide public health concern and will continue to pose a major challenge for healthcare systems and the individual, since its prevalence is expected to almost double by 2030, mainly due to unhealthy lifestyles^(2)^. According to the International Diabetes Federation, there will be approximately 580 million people with T2DM by the year 2030^(3)^.

Researchers over the past decades have used a variety of techniques in order to build numerous predictive models for the risk of T2DM, mainly because the majority of T2DM cases occur years before the clinical diagnosis^(4)^ and also because T2DM is associated with multiple complications when left untreated. Most of these predictive models include socio-demographic, clinical and genetic data as the predictive variables, yielding high values of area under the receiver operating characteristic (ROC) curve (AUC) ranging from 0⋅60 to 0⋅91^(5)^. Moreover, over the past two decades, the issue of the importance of dietary habits in the prevention and management of T2DM has received considerable critical attention^(6)^, concluding that dietary changes, combined with lifestyle changes, can significantly reduce the incidence of T2DM^(7)^. However, the literature to date has not focused on incorporating food intake variables in predictive models for the risk of T2DM, which may increase the accuracy of these models considerably.

We, therefore, developed a new predictive model of T2DM, including only food intake variables, using cross-sectional data from the Cooperative Health Research in the Region of Augsburg (KORA) FF4 study population. The major objective of the present study was to determine the performance measures of this model.

## Methods

### Study population

**Fig. 1.**
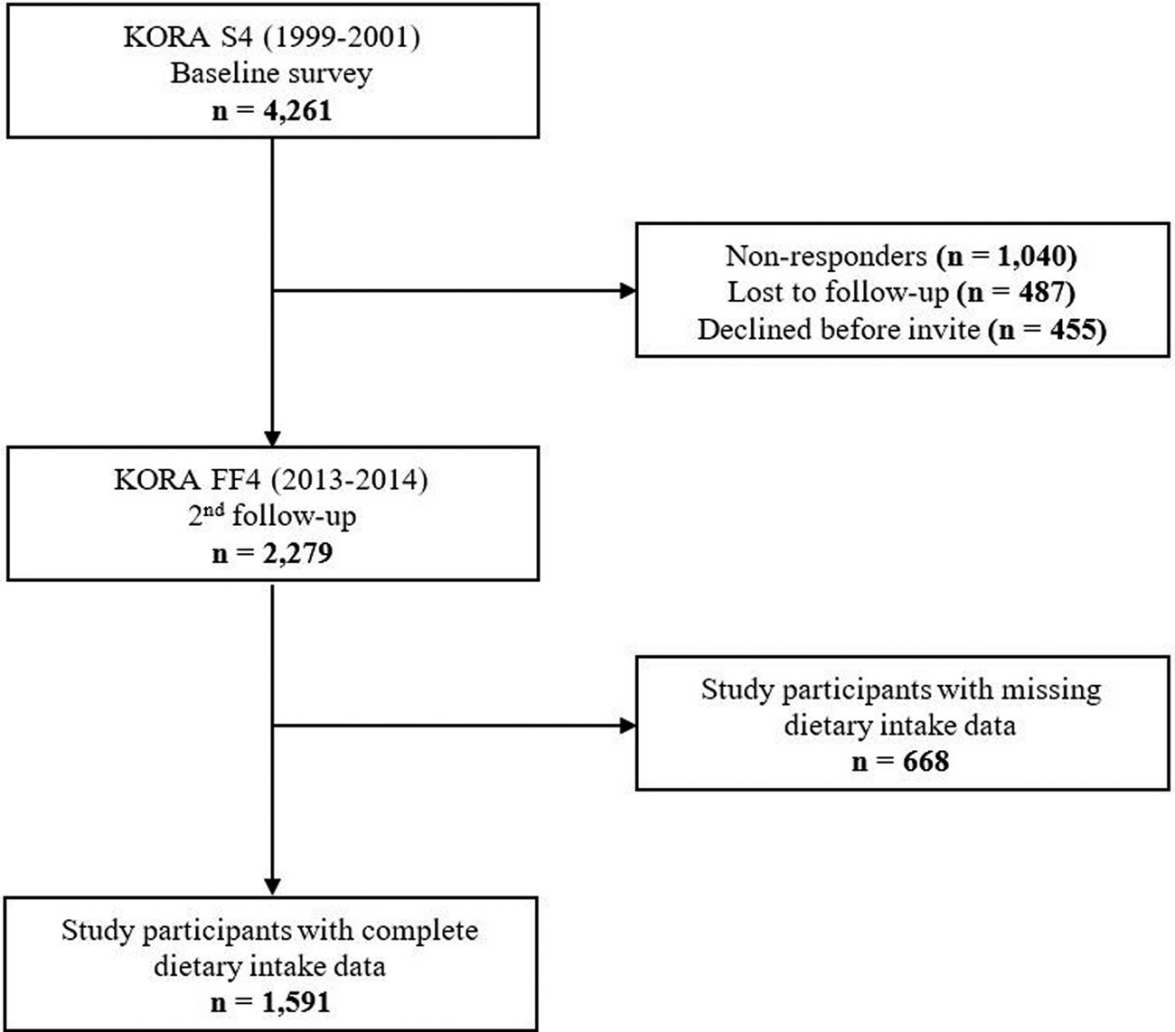
Flow diagram of study participants and exclusions in the Cooperative Health Research in the Augsburg Region (KORA) FF4 study.

The KORA FF4 study (2013–14) is the second follow-up examination of the population-based KORA S4 health survey, conducted in the city of Augsburg and two surrounding counties in Southern Germany between 1999 and 2001. Of all 4261 individuals included in the S4 baseline study, 2279 individuals also participated in the KORA FF4 study. The participation rate for KORA FF4 has been described previously^(8)^. Of the total 2279 participants, 668 were excluded from the analyses due to missing dietary intake data, resulting in 1591 individuals with complete data (Fig. 1).

The present study was carried out according to the Declaration of Helsinki. All examinations were approved by the ethics committee of the Bavarian Chamber of Physicians, Munich. Written informed consent was obtained from all participants.

### Assessment of habitual dietary intake

KORA FF4 dietary intake data was collected through one food frequency questionnaire (FFQ) and up to three 24-h food lists (24HFL). The FFQ was based on the German version of the multilingual European Food Propensity Questionnaire^(9)^ and assessed how frequently and in what amount 148 food items were consumed over the previous 12 months. The 24HFL are structured questionnaires, which encompass more than 300 food items and are used to assess the type of food consumed during the last 24 h. Details regarding the 24HFL have been published elsewhere^(10)^. The participants in KORA FF4 were asked to complete the first 24HFL at the study centre and the other two at home, preferably web-based in order to attenuate the presence of incomplete data; otherwise, a paper questionnaire could be provided. Finally, dietary intake data were available for 1591 participants who completed at least one 24HFL and one FFQ.

The usual dietary intake of all food times was calculated using a two-step approach, based on a combination of the National Cancer Institute method^(11)^ and the Multiple Source Method^(12)^. In the first step, the individuals’ consumption probability for each food item was calculated based on 24HFL dietary intake data, with logistic mixed models adjusted for age, sex, body mass index (BMI), physical activity, smoking, education and food consumption frequency calculated from the FFQ. In the second step, 24-h dietary recall data from the Bavarian Food Consumption Survey II^(13)^ were analysed in order to calculate the consumption amount of each food item, since the 24HFL do not assess portion sizes. Mixed models containing the same covariates as in the first step were used, and the usual consumption amount of each food item was predicted from the β-estimates of these models. The product of the estimated consumption probability and estimated consumption amount of each food item provided the usual dietary intake of all food items on a consumption day. Finally, the dietary intake data were divided into sixteen food groups based on the European Prospective Investigation into Cancer and Nutrition Soft classification system^(14)^. Data from the German Food Composition Database were used to estimate nutrient intakes (Bundeslebensmittelschlüssel BLS3.02). In total, 193 dietary intake variables were included in the analysis after the removal of the food variables that were registered as ‘other and unclassified’.

### Ascertainment of T2DM, age and sex

In addition to the habitual food intake variables, age, sex, BMI and T2DM status were included in the analysis. T2DM was assessed through self-reports of the participants and also by the current use of antidiabetic medication, both of which were verified by the individuals’ treating physician. The age and sex of participants were assessed through questionnaires and a computer-assisted personal interview. The BMI of participants was calculated by trained staff performing anthropometric measurements of height and weight.

### Statistical analysis

We compared the sixteen main groups of food intake variables among study participants with and without diabetes using percentage values for categorical variables and medians (25th and 75th percentile) for continuous variables.

We constructed a predictive model of T2DM status using the elastic net penalised multivariate regression method^(15)^, which combines the methods of lasso and ridge regression. We used the R package ‘glmnet’, where the elastic net algorithm is implemented in order to build our model^(16)^. This method is suitable for creating predictive models using datasets where collinear predictors are present, like in our case. The elastic net model requires two parameters (*α* and *λ*) to be specified and also needs its variables to be scaled. *α* indicates the ratio of the two penalties (*L*_1_ and *L*_2_), while *λ* is the shrinkage parameter. The optimal value of the *λ* parameter was selected using 5-fold cross-validation. After fitting the elastic net regression model in the training dataset, the model is fitted in the test dataset in order to calculate the performance of the model by the AUC. This process is repeated five times until every subset of the dataset has been a test dataset. In this way, the optimal value of *λ*, which produces the model with the highest AUC, can be calculated. Since a function to calculate the optimal value of the *α* parameter is not implemented in the ‘glmnet’ package, we calculated the optimal value of the *λ* parameter at six fixed different values of *α*: 0, 0⋅2, 0⋅4, 0⋅6, 0⋅8 and 1, respectively.

To identify the significant regression coefficients of the food intake variables, we used Bunea *et al.*'s approach^(17)^, by implementing bootstrapping in our analysis. We created 5000 bootstrap samples, by sampling our dataset with replacement 5000 times. Then, we built a predictive model for each bootstrap sample, and hence, the percentage of times each predictor was non-zero was calculated, which is called the variable inclusion probability (VIP). We used the open-source R code that is annotated in the study of Abram *et al.*^(18)^ in order to make the analysis and produce the graph with the selected variables at different thresholds of the VIP.

Finally, after selecting the food intake variables with standardised regression coefficients which were non-zero in more than 95 % of all bootstraps, we fitted a logistic regression model with these predictors and calculated its value of the AUC. We also calculated the values of specific performance measures of our model: sensitivity, specificity, positive predictive value (PPV), negative predictive value (NPV) and accuracy. *P*-values <0⋅05 were considered statistically significant. All analyses were carried out using the statistical software R (version 3.5.1, Foundation for Statistical Computing, Vienna, Austria).

## Results

From a total number of 1591 participants, 139 (8⋅7 %) were diagnosed with T2DM. Individuals with T2DM were more likely to be older, to be male, to have higher BMI and to have a higher intake of potatoes and meat products and a lower intake of vegetables, pulse, dairy products, sweets and non-alcoholic drinks (Table 1).

**Table 1.** Characteristics of the study population by type 2 diabetes status^a^

	Non-diabetics (*N* 1452)	Diabetics (*N* 139)	*P*-value
Age (years)	59⋅0 [49⋅0, 68⋅0]	72⋅0 [64⋅0, 76⋅0]	<0⋅001
Female (%)	53⋅2 [772]	39⋅6 [55]	0⋅003
BMI (kg/m^2^)	27⋅3 [24⋅1, 29⋅8]	30⋅8 [27⋅0, 34⋅3]	<0⋅001
Potatoes (g/d)	55⋅2 [44⋅2, 69⋅8]	63⋅8 [52⋅2, 83⋅0]	<0⋅001
Vegetables (g/d)	165 [134, 207]	150 [125, 192]	0⋅007
Pulse (g/d)	4⋅85 [3⋅70, 7⋅00]	4⋅50 [3⋅55, 5⋅90]	0⋅039
Fruits (g/d)	159 [100, 225]	166 [118, 228]	0⋅225
Dairy products (g/d)	181 [117, 263]	151 [97⋅7, 211]	<0⋅001
Cereal products (g/d)	161 [133, 195]	157 [130, 184]	0⋅177
Meat products (g/d)	105 [82⋅0, 141]	121 [94⋅8, 151]	<0⋅001
Fish and shellfish (g/d)	16⋅6 [11⋅9, 24⋅8]	16⋅8 [12⋅3, 24⋅8]	0⋅494
Egg products (g/d)	13⋅6 [10⋅2, 19⋅3]	14⋅5 [11⋅4, 19⋅5]	0⋅094
Fats (g/d)	23⋅7 [18⋅9, 30⋅0]	25⋅8 [19⋅6, 31⋅2]	0⋅061
Sweets (g/d)	35⋅8 [27⋅0, 47⋅1]	28⋅9 [21⋅6, 38⋅2]	<0⋅001
Cakes (g/d)	49⋅3 [38⋅7, 64⋅7]	50⋅0 [39⋅5, 63⋅9]	0⋅484
Non-alcoholic drinks (g/d)	1537 [1379, 1727]	1493 [1309, 1692]	0⋅043
Sauces (g/d)	22⋅4 [19⋅3, 26⋅9]	22⋅5 [19⋅6, 25⋅2]	0⋅440
Soups (g/d)	3⋅30 [2⋅20, 5⋅20]	3⋅20 [2⋅30, 5⋅30]	0⋅526
Other (g/d)	6⋅70 [4⋅90, 10⋅0]	6⋅40 [5⋅00, 10⋅0]	0⋅827

a Medians and 25th and 75th percentiles for continuous variables; percentages and counts for categorical variables.

Supplementary Fig. S1 of Supplementary material displays the boxplots of the 5000 calculated AUC values, derived from the 5-fold cross-validation procedure, for each *α* value. The mean values of the AUC values remain relatively stable across the six fixed *α* values, so we chose the lasso model which is the most parsimonious (*α* = 1). In Supplementary Fig. S2 of Supplementary material, significance thresholds are plotted, at which each of the 193 food intake variables is declared significant using the VIP approach. The grey bar of each predictor specifies the maximum value of the significance threshold that will result in the inclusion of each predictor in the final model. In simpler words, the grey bar indicates the percentage of non-zero coefficient values in the 5000 samples, for each predictor. It is also clear from the plot that when the elastic net model with *α* 0 is fitted (ridge regression), all the coefficients are present at every bootstrap sample, since ridge regression does not zero out any coefficients.

**Fig. 2.**
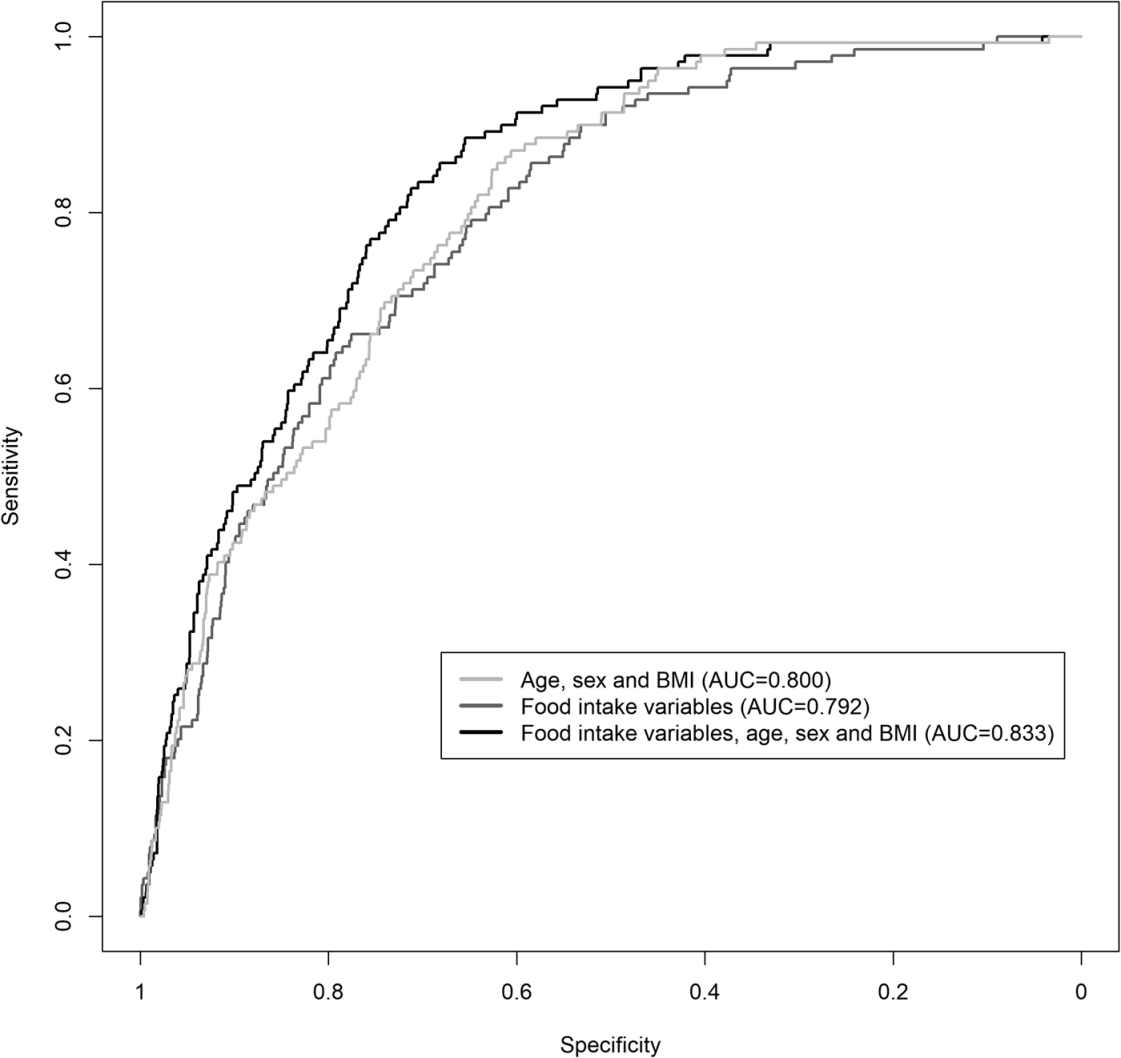
ROC curves of the predictive logistic regression models of T2DM using food intake variables, age, sex and BMI. AUC: area under the ROC curve; T2DM, type 2 diabetes mellitus.

By applying the VIP threshold of 95 %, eleven food intake variables were selected. After fitting a logistic regression model with the selected variables, the AUC was 0⋅792 (95 % CI 0⋅756, 0⋅826) (Fig. 2), and the values of its performance measures at different sensitivity thresholds are listed in Table 2. The variables, which had the strongest positive effect on the odds of having T2DM, were potatoes, xylitol, onions and garlic, and soft drinks, while the strongest negative effect was exerted from tomato sauce and mushrooms. Specifically, one standard deviation increases in potatoes, xylitol, onions and garlic, and soft drinks were related with 32⋅8 % (95 % CI 12⋅0 %, 57⋅3 %), 28⋅6 % (95 % CI 7⋅4 %, 54⋅1 %), 25⋅7 % (95 % CI 6⋅5 %, 48⋅5 %) and 25⋅0 % (95 % CI 6⋅4 %, 46⋅8 %), respectively, higher odds of T2DM, whereas one standard deviation increases in tomato sauce and mushrooms were related with 51⋅4 % (95 % CI 29⋅3 %, 66⋅6 %) and 68⋅6 % (95 % CI 48⋅9 %, 80⋅7 %), respectively, lower odds of T2DM.

**Table 2.** Performance measures of the predictive model for the risk of type 2 diabetes at different sensitivity thresholds^a^

Sensitivity	Specificity	PPV	NPV	Accuracy
0⋅1	0⋅98	0⋅37	0⋅92	0⋅91
0⋅2	0⋅96	0⋅33	0⋅93	0⋅89
0⋅3	0⋅93	0⋅29	0⋅93	0⋅87
0⋅4	0⋅91	0⋅29	0⋅94	0⋅86
0⋅5	0⋅86	0⋅26	0⋅95	0⋅83
0⋅6	0⋅81	0⋅23	0⋅95	0⋅79
0⋅7	0⋅73	0⋅20	0⋅96	0⋅73
0⋅8	0⋅63	0⋅17	0⋅97	0⋅80
0⋅9	0⋅53	0⋅16	0⋅98	0⋅90

a Only food intake variables are included in the model as predictive variables.

NPV, negative predictive value; PPV, positive predictive value.

By adding age, sex and BMI together with the food intake variables in our predictive model, the AUC increased slightly to 0⋅833 (95 % CI 0⋅801, 0⋅862) (Fig. 2). The odds ratio per one standard deviation increment for the selected food intake variables with their corresponding 95 % CI is shown in Table 3.

**Table 3.** Results of the multivariate logistic regression analysis with the selected food intake variables as predictors of type 2 diabetes

Food intake variables	Odds ratios per one standard deviations increment	95 % confidence intervals	*P*-value
Potatoes	1⋅328	1⋅120, 1⋅573	0⋅001
Mushrooms	0⋅314	0⋅193, 0⋅511	<0⋅001
Onions and garlic	1⋅257	1⋅065, 1⋅485	0⋅007
Whipping cream	1⋅194	1⋅031, 1⋅382	0⋅018
Cookies	1⋅111	0⋅920, 1⋅341	0⋅275
Soft drinks	1⋅250	1⋅064, 1⋅468	0⋅006
Coffee	0⋅736	0⋅570, 0⋅951	0⋅019
Tomato sauce	0⋅486	0⋅334, 0⋅707	<0⋅001
Mayonnaise	0⋅772	0⋅545, 1⋅094	0⋅145
Xylitol	1⋅286	1⋅074, 1⋅541	0⋅006
Galactose	0⋅713	0⋅563, 0⋅903	0⋅005

## Discussion

The present study was set out with the aim of assessing the importance of nutrition data in predictive modelling for the risk of T2DM. Thus, we incorporated various food variables in a predictive model for T2DM and calculated its performance measures. In our analysis, using the population-based cross-sectional data from the KORA FF4 study, we built a predictive model for the risk of T2DM, which yielded an AUC of 0⋅792.

A considerable amount of literature has been published on prediction models for T2DM. There was an exponential upward trend in the number of studies introducing new prediction models from 2006 and onwards^(5)^, providing good estimates on the risk of having or developing T2DM in the future. A wide set of techniques was used in order to build these models, including also in the last years’ methods developed from the computer science field, like neural networks^(19)^, which have been shown to increase the prediction accuracy of these models. Moreover, all these predictive models presented in the current literature were based on socio-demographic, clinical and/or genetic data, with the latter influencing slightly the performance of the models^(5,20)^. The most commonly used predictors for the risk of T2DM are age, family history of diabetes, BMI, hypertension, waist circumference, sex, ethnicity and smoking status^(20)^.

Despite the constant increase in developing new predictive models for T2DM within the last years, there have been reported only a few studies involving nutrition data in their analysis in the form of T2DM risk scores^(21–23)^. Over the past two decades, numerous studies highlighted the importance of lifestyle factors, including physical activity, weight loss, nutrition and smoking, in the prevention and management of T2DM^(24–26)^. With regard to nutrition, it has been found that specific dietary behaviours reduce significantly the incidence of T2DM^(26,27)^ and others can increase it^(28)^, and so including nutrition data into the T2DM risk prediction models could improve their performance. The present results, and specifically the performance measures of our model, highlight the important role that nutrition can play in identifying individuals with an increased risk for T2DM.

There are several possible explanations for these results. First of all, specific dietary patterns may influence the individuals’ risk of T2DM, since they can have a significant role in the pathogenesis of this chronic disease. For example, plant-based diets have been reported to reduce the risk of T2DM by jointly improving insulin sensitivity, controlling weight gain, resulting in weight loss and lowering systemic inflammation^(27)^. On the other hand, specific food groups, like red and processed meat, are related to increased T2DM risk^(28)^, possibly due to high haem iron and dietary cholesterol concentration^(29)^. Second, specific food intake variables can be related to clinical and socio-demographic factors, like BMI, age and sex^(30,31)^, which have been previously reported to act as strong predictors of the T2DM risk. This can also explain the similar values of AUC between the predictive model of T2DM status with only food intake variables included and the one with only age, sex and BMI (Fig. 2). The cross-sectional study design may have also been partly responsible for this small difference, since the real performance measures of our predictive model can be validated only under a longitudinal design. However, given the increasing prevalence of T2DM^(3)^, even small improvements on the AUC of T2DM predictive models may have a significant effect on the amount of people that could eventually be identified from these models as high risk for developing T2DM. Third, certain diet patterns have been found to have an effect on the control and management of T2DM^(32)^. So, owning to the cross-sectional nature of our data, it may also be possible that specific food intake variables can be strong predictors of T2DM, because diabetic individuals may have formed different dietary habits from non-diabetics, as a part of their disease management. The reverse association between T2DM and food intake variables can also explain some of the association estimates in the present study. For example, garlic supplement has been found to control blood glucose in T2DM patients^(33)^ and may be part of the T2DM therapy for many of them. However, the association estimate of garlic intake with T2DM was positive in the present study, which could have been due to a probable reverse association of T2DM with garlic intake and the predictive and not causal nature of our statistical model. In these ways mentioned earlier, the performance of our predictive model can be explained.

The present results have some important implications for public health practice and clinicians. The implementation of nutrition data into new predictive models for T2DM may create new risk scores that will be able to identify early people who will develop T2DM and so suggest them medical consultation. Moreover, the assessment of patients’ nutrition based on self-report is relatively easy to be done. Hence, although the incorporation of a predictive model into clinical practice is usually hard and influenced by multiple contextual factors, including nutrition data into predictive models may be feasible.

Strengths of the present study include its population-based design, large sample size, objective assessment of T2DM and assessment of a full range of food items. However, several limitations need to be taken into consideration. First, we were not able to assess the temporal ordering of the consumption of the food variables and T2DM because of the cross-sectional study design. Second, the assessment of the food intake variables was based on self-report, so measurement error cannot be excluded. Third, we have not validated our prediction model in an external dataset. Lastly, we did not include important predictors, and the most commonly used, for the risk of T2DM include family history of diabetes, physical inactivity, history of hyperglycaemia and use of anti-hypertensives^(20)^ and we tried, instead, to focus mainly on the predictive performance of food intake variables. Despite having probably undermined the performance measures of our model by excluding such important predictors of T2DM, food intake variables alone in our predictive model yielded a high AUC and similar to that of the predictive model with only age, sex and BMI included. Thus, we believe that furtherly examining the predictive and causal effect of food intake variable under explanatory studies could help us manage T2DM in a better way, regarding the screening, prevention and treatment of T2DM patients, and that was the main reason for building a predictive model with only food intake variables.

In conclusion, we found that building a predictive model of T2DM risk with including only food intake variables can yield high-performance measures. Implementing nutrition data into predictive models that will be used by clinicians and public health practitioners may be feasible. However, further population-based longitudinal studies, which will take these variables into account, are needed to confirm the present results.

## Supplementary material

For supplementary material accompanying this paper visit https://doi.org/10.1017/jns.2021.36. click here to view supplementary material
