# Supplementary material for: Prediction of type 2 diabetes mellitus based on nutrition data
Source: J Nutr Sci. 2021 Jun 21;10:e46. doi: 10.1017/jns.2021.36 (PMC8223171; doi:10.1017/jns.2021.36)
Supplement: Supplementary file 1 [file S2048679021000367sup001.rtf]

Supplementary Figure 1. Boxplot of the area under the ROC curve values using 5,000 bootstrap samples a
a Alpha: the ratio of the two penalties (L1 and L2) in elastic net regression; AUC: area under curve; ROC: receiver operating characteristic


Supplementary Figure 2. The significance threshold for all the 193 food intake variables at 6 different alpha values of the elastic net model a


a Threshold: percentage of the standardized regression coefficients of each food intake variable, which was non-zero in the bootstrap samples; á: the ratio of the two penalties (L1 and L2) in elastic net regression
